# *Dazl* Functions in Maintenance of Pluripotency and Genetic and Epigenetic Programs of Differentiation in Mouse Primordial Germ Cells *In Vivo* and *In Vitro*

**DOI:** 10.1371/journal.pone.0005654

**Published:** 2009-05-21

**Authors:** Kelly M. Haston, Joyce Y. Tung, Renee A. Reijo Pera

**Affiliations:** Institute for Stem Cell Biology & Regenerative Medicine, Department of Obstetrics and Gynecology, Stanford University School of Medicine, Palo Alto, California, United States of America; Baylor College of Medicine, United States of America

## Abstract

**Background:**

Mammalian germ cells progress through a unique developmental program that encompasses proliferation and migration of the nascent primordial germ cell (PGC) population, reprogramming of nuclear DNA to reset imprinted gene expression, and differentiation of mature gametes. Little is known of the genes that regulate quantitative and qualitative aspects of early mammalian germ cell development both *in vivo*, and during differentiation of germ cells from mouse embryonic stem cells (mESCs) *in vitro*.

**Methodology and Principal Findings:**

We used a transgenic mouse system that enabled isolation of small numbers of *Oct4*ΔPE:GFP-positive germ cells *in vivo*, and following differentiation from mESCs *in vitro*, to uncover quantitate and qualitative phenotypes associated with the disruption of a single translational regulator, *Dazl*. We demonstrate that disruption of *Dazl* results in a post-migratory, pre-meiotic reduction in PGC number accompanied by aberrant expression of pluripotency genes and failure to erase and re-establish genomic imprints in isolated male and female PGCs, as well as subsequent defect in progression through meiosis. Moreover, the phenotypes observed *in vivo* were mirrored by those *in vitro*, with inability of isolated mutant PGCs to establish pluripotent EG (embryonic germ) cell lines and few residual *Oct-4*-expressing cells remaining after somatic differentiation of mESCs carrying a *Dazl* null mutation. Finally, we observed that even within undifferentiated mESCs, a nascent germ cell subpopulation exists that was effectively eliminated with ablation of *Dazl*.

**Conclusions and Significance:**

This report establishes the translational regulator *Dazl* as a component of pluripotency, genetic, and epigenetic programs at multiple time points of germ cell development *in vivo* and *in vitro*, and validates use of the ESC system to model and explore germ cell biology.

## Introduction

In mice, the formation of the germ line is first noted at approximately embryonic stage (E) 6.25, when a population of ∼6 cells in the proximal epiblast first express the *Blimp*-1 gene in response to inductive signaling from extraembryonic ectoderm [1]–[5]. Following specification, the founder population gives rise to ∼40 PGCs which migrate out of the embryo to reside in extraembryonic tissues until gastrulation is complete. Subsequently, the cells migrate to the nascent gonads and proliferate while they begin the process of erasure of genomic imprints (E9.5–11.5) [6]–[8]. Following differentiation of the gonads to testes or ovaries, germ cell sex is determined to be male or female and diagnostic sex-specific imprinted gene methylation patterns are established [9], as development diverges and male PGCs mitotically arrest while female PGCs enter meiosis [10].

Throughout early development, PGCs express the germ cell specific gene *Dazl (Deleted in AZoospermia-Like)*, a member of the *DAZ* gene family, which encodes RNA-binding proteins required for germ cell development in diverse organisms [11]. DAZ family proteins interact with several other RNA binding proteins to regulate translation in diverse organisms [12]–[16]. A number of studies provide evidence that *Dazl* is required in diverse species for germ cell development [17]–[26]. Indeed, disruption of mouse *Dazl* leads to loss of germ cells in the gonads of both sexes though timing and quantification of loss has not been well established [22]. Furthermore, recent work reported the phenotype of mutant male mouse embryos at the histological level and demonstrated an increased number of apoptotic germ cells, reduced expression of some germ cell markers, and a chromatin configuration typical of immature germ cells prenatally in the absence of *Dazl* function (on a C57BL/6 background) [26]. However, to date, methods have not allowed examination of isolated PGCs at multiple stages of development both *in vivo* and *in vitro* leading to reports of distinctly different phenotypes.

Concurrently, several reports have documented the *in vitro* differentiation of female (XX) and male (XY) germ cells from mouse embryonic stem cells (mESCs; [27]–[31]). Presumptive germ cells were identified via a combination of distinct morphological characteristics, germ cell-specific gene expression profiles, and diagnostic changes in epigenetic methylation status at imprinted loci. Similarly, human embryonic stem cells (hESCs), and those of non-human primates, were shown to differentiate to the germ cell lineage with increased numbers of germ cells obtained with addition of factors such as BMP4, BMP7 and BMP8 [32]–[34]. Importantly, studies of germ cell development *in vitro* routinely include *Dazl* as a definitive early marker of mouse and human germ cell development [29]–[35]. Thus, diverse mammalian ESC lines appear to possess ability to differentiate to the germ cell lineage, potentially providing a tractable *in vitro* system to study germ cell development. Yet a major limitation remains with the lack of evidence to correlate differentiation *in vitro* with landmark events and genetic requirements *in vivo*.

Here, we compared genetic requirements for germ cell development *in vitro* and *in vivo*. For this purpose, we derived wildtype, heterozygous *Dazl+/−* and *Dazl−/−* null mESC lines and directly compared germ cell differentiation, *in vivo* and *in vitro*, based on knowledge of landmark events and diagnostic gene expression patterns (Figure S1; adapted from [34]).

## Results

### *Dazl* is Required for Germ Cell Maintenance Prior to E14.5

The *Oct4*ΔPE:GFP transgene has been reported to be expressed exclusively in PGCs *in vivo* throughout fetal germ cell development in both males and females [36]–[40]. We verified that Oct4ΔPE:GFP expression occurs only in germ cells and not in somatic cells of wildtype and *Dazl*−/− mutant mice as shown (Figures S2A–S2D). In addition, we compared gene expression in GFP-positive and GFP-negative cells and observed that only cells positive for GFP expressed *Oct4* and other germ cell-specific markers such as *Vasa*, while the GFP-negative population was enriched for somatic markers such as α-fetoprotein (Figure S2 E).

**Figure 1:**
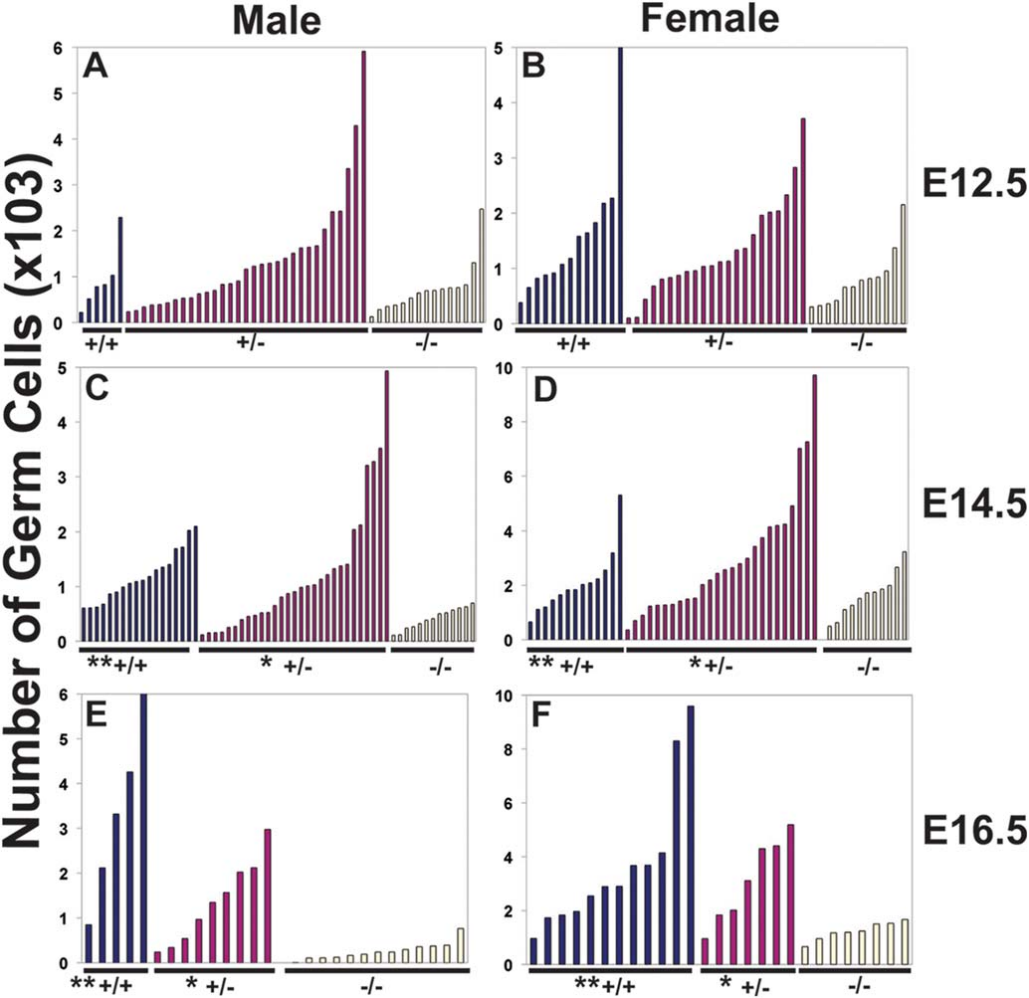
Requirement for *Dazl* in Embryonic Germ Cells. (A) Total germ cell counts in E12.5 testis. (B) Total germ cell counts in E12.5 ovary. (C) Total germ cell counts in E14.5 testis. (D) Total germ cell counts in E14.5 ovary. (E) Total germ cell counts in E16.5 testis. (F) Total germ cell counts in E16.5 ovary. Number of germ cells is on the y-axis. ‘*’ denotes a statistically significant difference in germ cell count with the *Dazl−/−* mutant (p<0.05). ‘**’ denotes a statistically significant difference in germ cell count with the *Dazl−/−* mutant (p<0.001).

We next crossed mice heterozygous for the *Dazl* null allele [22] to mice carrying the Oct4ΔPE:GFP transgene [40] to produce wildtype, heterozygous, and *Dazl* null mice with germ cells that express the GFP transgene. Embryonic gonads were dissected from these mice and germ cells were counted by fluorescent-activated cell sorting (FACS) at E12.5, E14.5, and E16.5 (Figures 1A–1F). We observed substantial variation in native germ cell numbers between different mice, especially in early development; this natural variation likely reflects a combination of differences in the timing of the completion of migration, proliferation of initial germ cell populations, and arrest or entry into meiosis, as well as genetic background effects as previously reported [2], [41]–[43].

To test the significance of differences in germ cell number amongst genotypes, we fit the data to a negative binomial regression and stratified the data by litter. At E12.5, the *Dazl* null mice appeared to have fewer germ cells; however differences were not significant amongst the different genotypes in either sex (Figures 1A–1B). In contrast, by E14.5, the number of germ cells was significantly different in homozygous *Dazl−/−* mutants relative to both wildtype mice and heterozygous *Dazl+/−* mice (Figures 1C–1D). Differences were magnified further at E16.5, with a significant deficiency in germ cell numbers in the null mutant relative to both heterozygous and wildtype genotypes in both male and female embryos (Figures 1E–1F). We observed no significant differences in germ cell numbers in heterozygous embryos relative to wildtype.

### *Dazl* Mutants and Germ Cell Proliferation and Apoptosis

Since we observed that the number of germ cells was significantly reduced in *Dazl−/−* mutant mice by E14.5, we next examined germ cell proliferation and apoptosis a day earlier (at E13.5) to assess contribution to the reduced germ cell numbers. Gonads from E13.5 Oct4ΔPE:GFP-positive embryos were stained with propidium iodide (PI) to mark DNA content and analyzed by FACS. Although variation in germ cell numbers was observed as previously, we did not observe any significant or consistent differences across genotypes in the percentage of cells in G1 (with 2N DNA content) or G2/M (with 4N DNA content) in male and female *Dazl−/−* mutants relative to wildtype littermates (Figure S3). We also examined isolated germ cells for evidence of apoptosis by staining with DAPI and counted the number of germ cells with canonical fragmented nuclear morphology associated with apoptosis (data not shown). Although we observed a slight increase in the number of apoptotic cells in both the male and female *Dazl* mutant mice at E13.5 compared to the heterozygous and wildtype mice, given the very modest effects of both proliferation and apoptosis, we could not definitively conclude that either process, alone or in combination, leads to the reduced germ cell numbers.

### *Dazl* Mutants and Aberrant Germ Cell Specific Gene Expression

**Figure 2:**
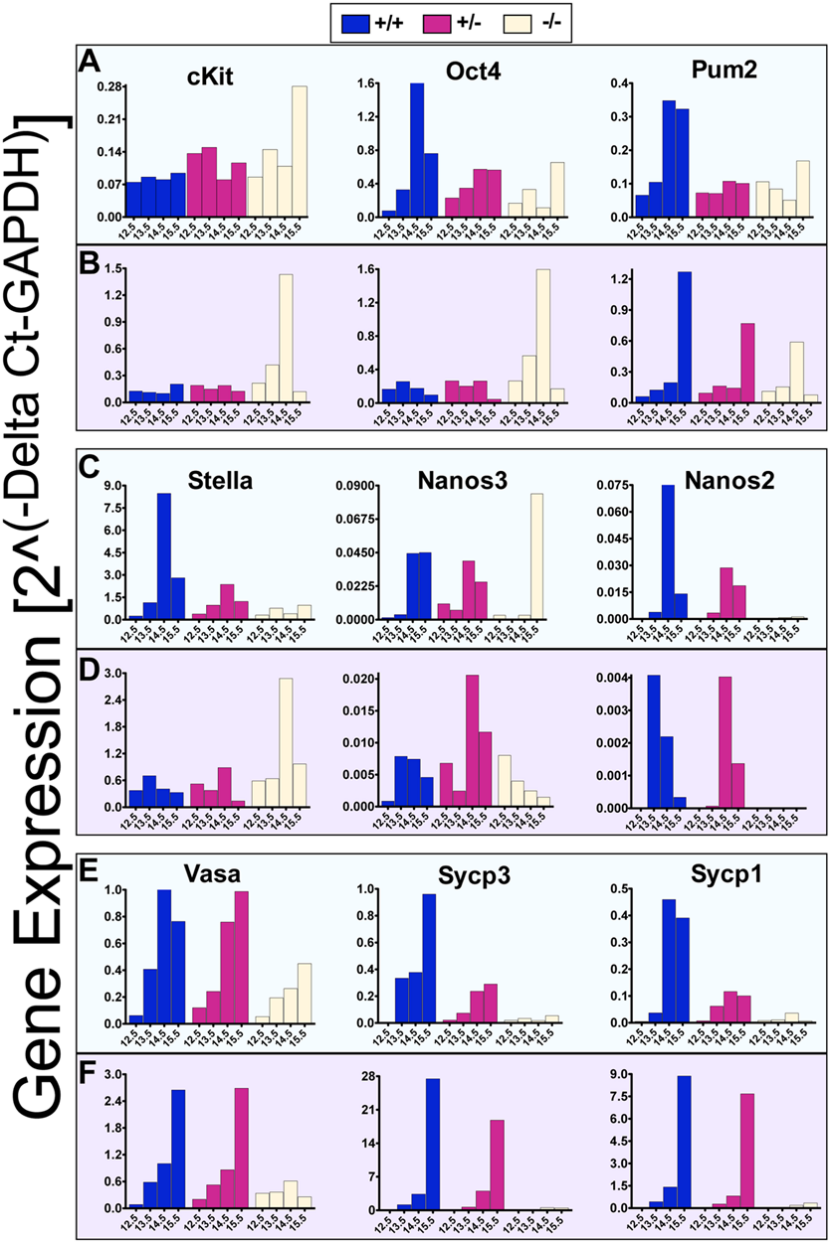
Aberrant Germ Cell Gene Expression in the Germ Cells of *Dazl* Mutants. Expression profiles are shown in isolated germ cells at E12.5, E13.5, E14.5, and E15.5. Males are displayed in green boxes (A), (C), and (E). Females are displayed in purple boxes (B), (D), and (F). cKit, Oct4, Pum2 (Pumilio2), Stella, Nanos3, and Nanos2 represent genes, which are expressed beginning in migrating PGCs, while Vasa, Sycp3 (Synaptonemal complex protein 3), and Sycp1 (Synaptonemal complex protein 1) represent meiotic markers. Expression levels on the y-axis are normalized to the *GAPDH* housekeeping gene. Each bar on the graph represents average expression of sorted germ cells from the following number of embryos. Males: E12.5 +/+ (N = 3), +/− (N = 13), −/− (N = 5); E13.5 +/+ (N = 4), +/− (N = 8), −/− (N = 3); E14.5 +/+ (N = 2), +/− (N = 17), −/− (N = 3); E15.5 +/+ (N = 7), +/− (N = 10), −/− (N = 3). Females: E12.5 +/+ (N = 5), +/− (N = 7), −/− (N = 6); E13.5 +/+ (N = 3), +/− (N = 5), −/− (N = 4); E14.5 +/+ (N = 8), +/− (N = 15), −/− (N = 4); E15.5 +/+ (N = 7), +/− (N = 7), −/− (N = 4). Note the Y-axis on all graphs display different scales.

To further examine effects of loss of function of *Dazl*, we analyzed gene expression in isolated germ cells of different *Dazl* genotypes (Figures 2). The *Nanos2*, *Nanos3*, *Pumilio-2 (Pum2)*, *Stella*, *Oct-4*, and *c-kit* genes are expressed in the earliest stages of germ cell development in PGCs before they reach the presumptive gonad. Other genes, such as *Vasa*, *Sycp3* and *Sycp1*, are expressed in germ cells only after they reach the gonad and/or initiate meiosis (as reviewed in Figure S1). We observed clear differences in gene expression in germ cells of different genotypes beginning at E13.5 and extending through E15.5 in both sexes. In the male, the *Dazl−/−* mutant germ cells had decreased expression of early markers of pluripotent germ cells such as *Nanos2*, *Nanos3*, *Pum2*, *Stella*, and *Oct-4*, and even larger decreases in the expression of meiotic markers such as *Sycp3* and *Sycp1*, relative to the wildtype cells at E13.5, 14.5 and 15.5 (Figure 2A, 2C, and 2E). In contrast at E12.5, no differences in gene expression across genotypes were observed in male germ cells.

In females, a comparison of gene expression across genotypes indicated that *Dazl−/−* null germ cells expressed higher levels of several early markers, including *Stella*, *Oct-4*, and *c-kit* at E13.5 and E14.5, and to some extent at E12.5 relative to wildtype littermates (Figure 2B & 2D); indeed the elevated expression of the premeiotic marker, *Stella*, persisted until E15.5 (Figure 2D). In contrast, *Pum-2* expression in the mutant was significantly reduced at E15.5 (Figure 2B) and *Vasa* expression in the mutant germ cells decreased over time from E12.5 to almost negligible levels at E15.5 (Figure 2F). Beginning at E13.5 in females, *Dazl−/−* mutant germ cells also demonstrated much lower expression of the meiotic genes, *Sycp3* and *Sycp1*, relative to wildtype germ cells (Figure 2F). Notably, isolated female germ cells of all genotypes expressed much higher levels of these meiotic genes relative to male germ cells in keeping with the fact that female, but not male, germ cells enter meiosis during this developmental window.

### *Dazl* and Erasure and Re-establishment of H19 DMR Imprinting Methylation

We next explored whether the loss of *Dazl* function impacts erasure and re-establishment of genomic imprints, a functional landmark of nuclear reprogramming during germ cell development [44]–[45]. For this purpose, we focused on analysis of male germ cells due to the more contracted timeframe for erasure and subsequent re-establishment of imprints in male germ cells. In particular, the imprinting methylation marks at the H19 differentially methylated region (DMR) are re-established between E15.5 to E18.5 in males [46]–[49]. In contrast, methylation at imprinted loci is re-established over a more expanded time frame in female germ cells, continuing postnatally, making it difficult to assess methylation status in the few germ cells that may remain after fetal development.

**Figure 3:**
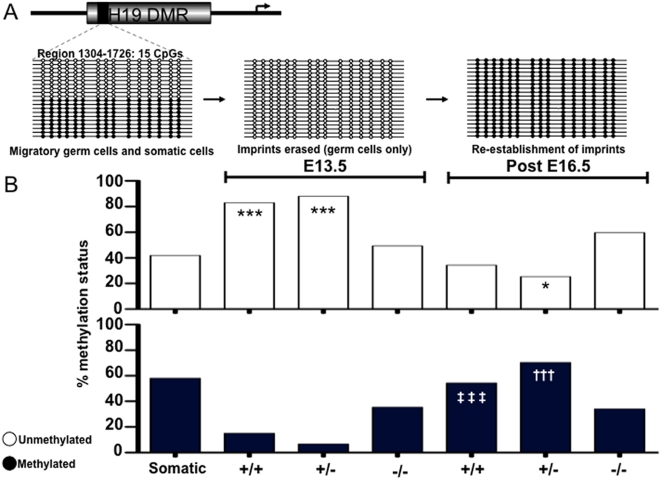
Methylation Marks at the H19 Imprinted Locus are disrupted in Germ Cells of *Dazl* Mutants. (A) Schematic of expected methylation status of 15 CpG islands in region 1304–1726 of the H19 differentially methylated region (DMR) (GenBank acc. No. U19619) at different time points in the lifecycle of male germ cell development. Open circles denote an unmethylated status of a CpG, while filled circles denote a methylated CpG. (B) Results of bisulphite sequencing displaying an average percent methylation status (y-axis) of 60 clones per datapoint (20 clones per individual sample, 3 replicates for each genotype/age) for both E13.5 and post E16.5 GFP-positive germ cells isolated by FACS. Clones that were hemi-methylated are not shown. ‘***’ denotes a very significant difference (P<0.001) in unmethylated status relative to somatic. ‘*’ denotes a statistically significant difference (P<0.05) in methylated status relative to somatic. ‘‡ ‡ ‡’ denotes an extremely significant difference (P<0.001) in methylated status with E13.5 D*azl*+/+. ‘†††’ denotes an extremely significant difference (P<0.001) in methylated status with E13.5 *Dazl*+/−.

Previous studies demonstrated that the methylation marks at the H19 locus are completely erased by E13.5 in male germ cells; subsequently, the male-specific methylation pattern is reestablished between E15.5 to E18.5. In contrast, somatic cells display a 1∶1 (50%) methylated∶unmethylated pattern throughout this developmental period, representing the contribution from each parental allele [46]–[49, outlined in Figure 3A].

As expected, somatic cells displayed a 1∶1 ratio of methylated and unmethylated H19 alleles (Figure 3B). We then examined the methylation status of H19 in germ cells from E13.5 embryos. As also expected, we observed that the methylation status in wildtype male germ cells differed substantially from that of somatic cells with only 15% of alleles methylated, fully 83% unmethylated (p<0.001), and the remainder hemi-methylated (characterized by incomplete methylation on a single strand, data not shown). This indicated that the majority of chromosomes in wildtype germ cell populations were in the process of erasing H19 methylation marks. Results from *Dazl* heterozygous male germ cells were similar to those from wildtype mice (6.7% methylated and 88% unmethylated, p<0.001). In contrast, however, at E13.5, erasure of methylation at the H19 locus was not observed in *Dazl*−/− mutant germ cells (methylation status of 35.4% methylated and 49.4% unmethylated; Figure 3B). Furthermore, although both wildtype and heterozygous embryonic germ cells had significantly different unmethylated status relative to somatic tissues, germ cells from *Dazl*-null embryos did not differ significantly in methylation status from somatic tissues. These data demonstrate that wildtype and heterozygous male germ cells have appropriately erased methylation marks at imprinted loci at E13.5, whereas erasure is delayed, or completely stalled, in *Dazl*−/− mutant germ cells.

Next, we examined methylation status at the H19 locus in germ cells obtained from E16.5 and neonatal mice to determine if sex-specific re-establishment of the H19 imprints in male germ cells had occurred. After confirming that there were no significant differences between late embryonic (E16.5) and neonatal averages (data not shown), we combined samples by genotype. Wildtype male germ cells displayed averages of 54.5% methylated and 34.4% unmethylated, while heterozygous males showed averages of 70.4% methylated and 25.4% unmethylated (p<0.05 relative to somatic; Figure 3B), and had significantly different methylation status in post-E16.5 samples relative to their respective E13.5 samples (p<0.001; Figure 3B). These results demonstrate that germ cells from both wildtype and heterozygous males are in the process of re-establishing H19 sex-specific methylation patterns. Again, in contrast, *Dazl*−/− germ cells showed averages of 34.1% methylated and 59.7% unmethylated, with no significant difference in imprinted methylation patterns between *Dazl*−/− mutant germ cells at days E16.5 and E13.5 (Figure 3B). Taken together, these data indicate that wildtype and heterozygous germ cells progress along a coordinated sequential program of erasure and re-establishment of H19 imprints in male germ cells, while in sharp contrast, the erasure and re-establishment of imprints in germ cells that lack *Dazl* function is aberrant, with both erasure and re-establishment of sex-specific imprints derailed. Thus, the loss of function of the *Dazl* translational regulator leads to reduced numbers of germ cells, altered expression of pluripotency genes, and failure to execute even the earliest stages of nuclear reprogramming at the H19 imprinted locus.

### *Dazl* Mutants Fail to Produce Embryonic Germ Cell Lines

**Figure 4:**
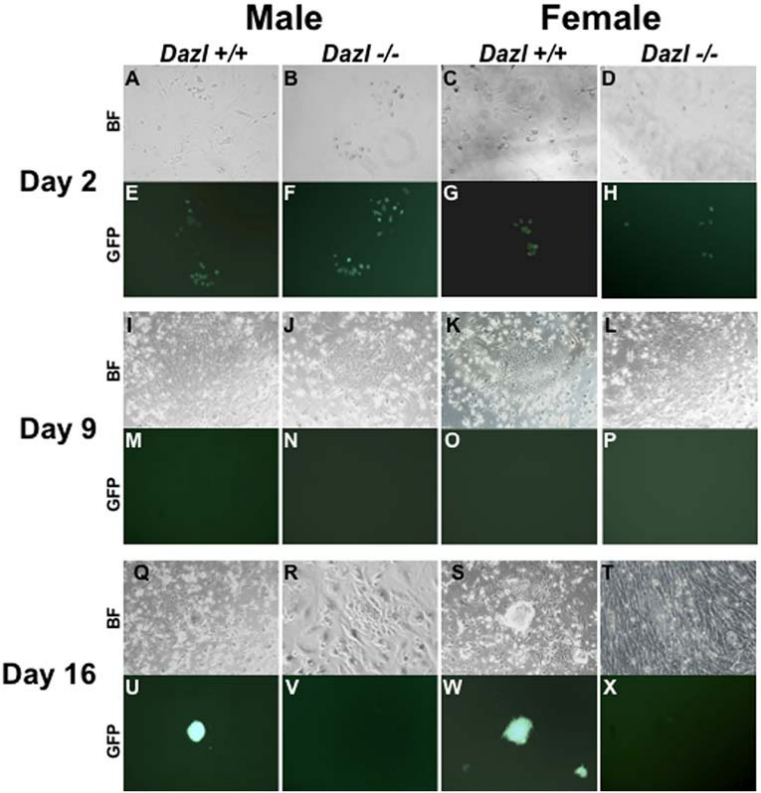
*Dazl* is required for Derivation of Embryonic Germ Cells Lines from E12.5 PGCs. Brightfield (BF) and GFP fluorescent images of Oct4ΔPE:GFP-positive PGCs during EG derivation on STO feeders from male and female E12.5 PGCs. (A–D) BF and (E–H) GFP expression of day 2 post-FACS wildtype and *Dazl* null PGCs. (I–L) BF of flattened, cobblestone like colonies at day 9 with no GFP expression (M–P). (Q–T) BF and (U–X) GFP expression in day 16 post-FACS cells, with only wildtype wells displaying EG colony formation (Q, S) and GFP expression (U, W).

To determine if PGCs from *Dazl* mutants were able to produce embryonic germ (EG) lines we dissected gonads from wildtype, *Dazl* heterozygous, and *Dazl* null E10.5, E12.5, E14.5, E15.5 and E16.5 male and female embryos and isolated the Oct4ΔPE:GFP-positive germ cells by FACS. We cultured the cells on STO-feeders in a PGC-defined media as previously described [50]. We did not derive EG lines from PGCs isolated from any genotype post-E12.5, as expected, or from the GFP-negative sorted fractions containing somatic gonadal cells. As displayed in Figure 4, GFP-positive cells isolated from wildtype and *Dazl* null PGCs at E12.5 gonads of both sexes express GFP at day 2 post-plating (Figure 4A–4H, heterozygous data not shown). However, by day 9, all wells, regardless of sex or genotype developed flattened colonies with a cobblestone-like appearance (Figure 4I–4L) and exhibit no GFP expression (Figure 4M–4P). By day 16 EG colony formation was apparent in both male and female wildtype samples, but not in *Dazl* null samples (Figure 4Q–4X; EG colonies were confirmed by alkaline phosphatase staining (data not shown)). We note that we saw a similar pattern of EG formation from wildtype male and female PGCs at E10.5 (data not shown), however, EG lines were never derived from *Dazl* heterozygous and *Dazl* null PGCs at any time point.

### *Dazl* and Meiosis

**Figure 5:**
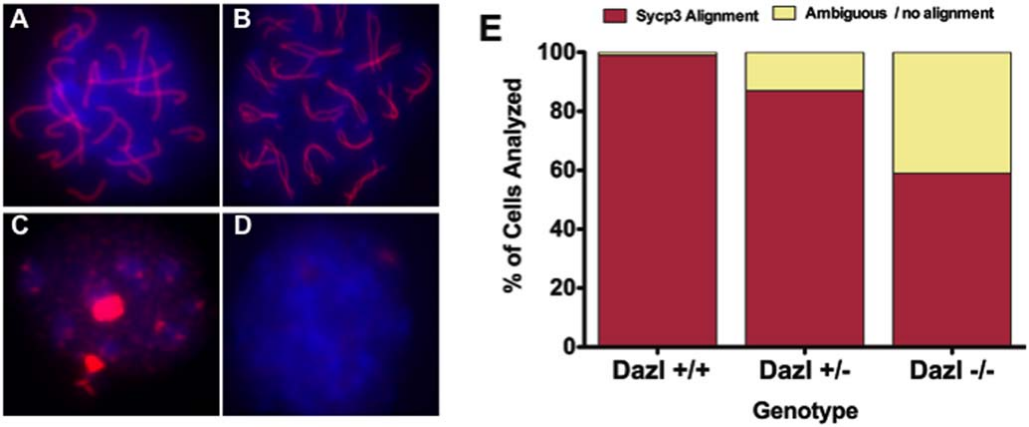
*Dazl* is required for Meiotic Progression, but Not for Entry into Meiosis. Results of analysis of 100 cells/genotype FACS isolated E17.5 Oct4ΔPE:GFP-positive cells, immunostained for SYCP3 (red) to indicate pachytene stage chromosomal alignment of Prophase I, and DAPI (blue) to label the nucleus. (A) Wildtype germ cell in pachytene. (B) *Dazl*−/− mutant germ cell in late zygotene/early pachytene. (C) *Dazl*−/− mutant germ cell displaying non-aligned SYCP3 staining. (D) *Dazl*−/− mutant germ cell displaying no SYCP3 staining. (E) Bar graph displaying percentage of cells per 100 cells/genotype showing appropriate SYCP3 alignment (A, B, red bars), and percentage of cells determined to be ambiguous or not showing SYPC3 alignment (yellow bars).

Next we isolated GFP-positive cells by FACS from E17.5 female embryonic gonads and immunostained with SYCP3 (Figure 5A–5D), to determine if germ cells carrying a *Dazl* null mutation enter and progress through meiosis. Our analysis of at least 100 cells/slide showed that 99% of germ cells isolated from wildtype gonads had SYCP3 alignment indicative of pachytene stage of prophase I of meiosis (5A, 5E). However, just 87% of germ cells isolated from *Dazl* heterozygous gonads were in pachytene stage (Figure 5E). Moreover, only 59% of *Dazl-null* germ cells had a similar SYCP3 alignment to wildtype (Figure 5B), while the remaining 31% did not display appropriate SYCP3 alignment, indicating that in the absence of *Dazl* function, a large percentage of female germ cells fail to appropriately progress through meiosis (Figure 5C–5E).

### Differentiation of Mouse ESCs to Germ Cells is Genetically Dependent on *Dazl*

A critical deficit in the use of *in vitro* differentiation to study early germ cell development is the lack of data to relate requirements *in vitro* to those *in vivo*. Thus, we sought to examine whether the requirement for *Dazl* function would be recapitulated in the differentiation of mESCs to germ cells *in vitro*. We mated 10 *Dazl* heterozygous couples, (copulation plug confirmed on the morning of E0.5), and isolated 66 blastocysts at E3.5. From these we obtained and characterized 44 unique mESC lines, most of which carried the *Oct4*ΔPE:GFP transgene, and were either wildtype, heterozygous, or homozygous null at the *Dazl* locus, with both sexes represented (Figure S4). We determined that the ratio of genotypes obtained from the 10 litters was very close to Mendilian ratios, although the *Dazl* null contribution was slightly lower than expected (Figure S5A). However, when we plotted the breeding results from our *in vivo* experiments we got the appropriate Mendilian ratios (Figure S5B), and these results together suggesting that *Dazl* ablation does not appear to affect genotype ratios during mESC line derivation. Furthermore, we performed metaphase spreads, and analyzed all female line to ensure that each maintained the correct karyotype of 40 chromosomes, with both X chromosomes represented (Figure S5C). Thereafter, for each gender/genotype combination we used 2 uniquely derived lines, with the exception of the female *Dazl* null sample, in which we were only able to derive one line that was *Oct4*ΔPE:GFP-positive, and used these 11 lines in all further experiments. Each line was differentiated in at least three independent experiments using a spontaneous differentiation protocol over a 35-day time course. Samples were analyzed by FACS at multiple timepoints for putative germ cell populations that retain *Oct4*ΔPE:GFP expression (results were averaged across gender/genotype combination after consideration of each line separately, data not shown). We hypothesized that if the *Oct4*ΔPE:GFP-positive population consisted of differentiated germ cells *in vitro*, we expected that loss of function of *Dazl* would reduce or eliminate *Oct4*ΔPE:GFP-positive cells relative to wildtype and/or heterozygous as observed *in vivo*.

**Figure 6:**
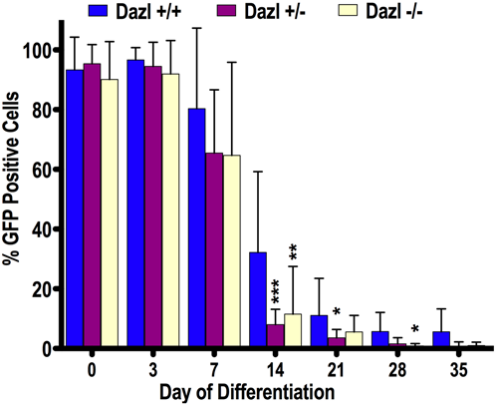
*Dazl* is required for *in vitro* Germ Cell Development. Counts of Oct4ΔPE:GFP positive cells in wildtype (+/+), *Dazl* heterozygotes (+/−), and *Dazl*-null (−/−) mutant cell lines during a 35-day embryoid body differentiation. Percent GFP positive of all live cells counted is on the y-axis. ‘***’ denotes an extremely significant difference (P<0.001) in percent GFP positive cells relative to wildtype. ‘**’ denotes a very significant difference (P<0.01) in percent GFP positive cells relative to wildtype. ‘*’ denotes a significant difference (P<0.05) in percent GFP positive cells relative to wildtype.

Our results indicated that at days 0 and 3, regardless of genotype or chromosomal sex, more than 90% of undifferentiated mESCs expressed GFP from the *Oct4*ΔPE:GFP promoter (Figure 6). In contrast to observations at days 0 and 3, we noted that by day 7 of differentiation, approximately 80% of wildtype cells were *Oct4*ΔPE:GFP-positive, whereas just 65% of differentiating cells from *Dazl+/−* and *Dazl*−/− mutant genotypes were *Oct4*ΔPE:GFP-positive. By day 14 of differentiation, the wildtype lines had an average of 32% *Oct4*ΔPE:GFP-positive cells, which was significantly different from the heterozygous average of 8% and the *Dazl-null* average of 11% *Oct4*ΔPE:GFP-positive cells (p<0.001 and p<0.01 respectively; Figure 6). Differences were observed throughout each subsequent timepoint, and by day 28, wildtype lines had an average *Oct4*ΔPE:GFP-positive population of approximately 5%, while both heterozygous and *Dazl-null* lines had an average *Oct4*ΔPE:GFP-positive population of less than 1%. Finally, there was near-complete absence of *Oct4*ΔPE:GFP positive cells in *Dazl*-null lines by day 35, an observation that strongly supports the concept that *in vitro* differentiation of germ cells parallels that *in vivo*.

### *Dazl* and Expression of Pluripotency and Germ Cell-Associated Genes *in vitro*

**Figure 7:**
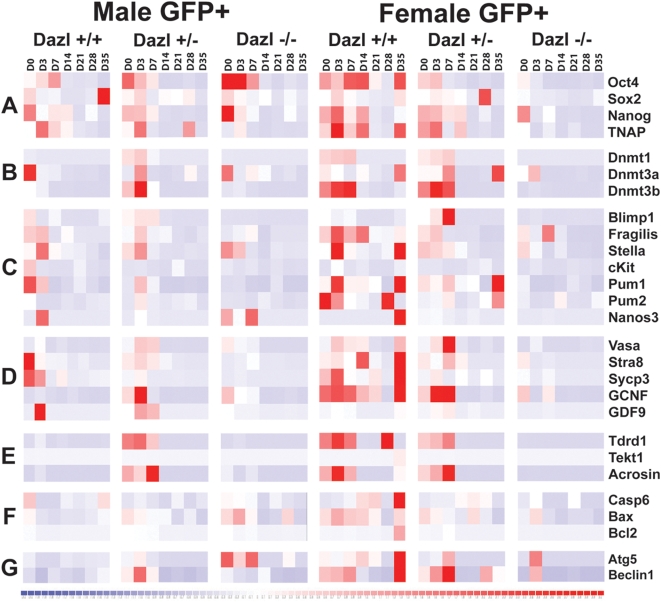
*Dazl* Mutant mESCs Display Aberrant Gene Expression of Pluripotency and Germ Cell Markers During Differentiation. Gene expression profiles are shown from GFP-positive cells, isolated by FACs, from day 0, 3, 7, 14, 21, 28, and 35 of differentiation. Expression is represented by heat map generated on DChip displaying a continuum with high expression depicted by red to low expression, in blue. The numeric values are from the formula 2∧^(−ΔCt)^, with GAPDH as the housekeeping gene, on a log scale. Genes are classified based by functional group as follows. Group A consisted of the pluripotency related genes *Oct4*, *Sox2*, *Nanog*, and TNAP (*Tissue-non-specific alkaline phosphatase*). Group B contains epigenetic regulators from the DNA methyltransferase family, *Dnmt1*, *Dnmt3a*, and *Dnmt3b*. Group C consists of the primordial germ cell specific markers *Blimp1*, *Fragilis*, *Stella*, *cKit*, *Pumilio1* (*Pum1*), *Pumilio2* (*Pum2*), and *Nanos3*; Group D contains the gonocyte markers *Vasa*, *Stra8*, *Sycp3 (Synaptonemal Complex Component 3)*, *GCNF*, and *GDF9*. Group E contains the late germ cell markers *Tdrd1* (*Tudor domain containing 1*), *Tekt1* (*tektin1*), and *Acrosin*; Group F contains pro-apoptotic markers *Casp6* (*Caspase6*) and *Bax* (*BCL2-associated X*), as well as the anti-apoptotic marker *Bcl*-2; Group G contains markers of autophagy including *Atg5* (*autophagy related* 5) and *Beclin1*.

We next examined gene expression in the *Oct4*ΔPE:GFP-positive cells isolated from differentiating mESCs using a quantitative PCR (qPCR) microfluidics approach to enable interrogation of many genes from very few cells. As stated above, we used 11 unique mESC lines and analyzed samples from 3 independent differentiation experiments on days 0, 3, 7, 14, 21, 28, and 35. Data is displayed in heat map format for clarity with relative expression on a continuum with greatest expression indicated by red, and lowest expression levels indicated by blue (Figure 7). We placed genes in categories to reflect gene expression levels and function (Figure 7 & Table S2). Group A contains the pluripotency related genes, *Oct4*, *Sox2*, *Nanog*, and TNAP (*Tissue-non-specific alkaline phosphatase*); Group B contains epigenetic regulators from the DNA methyltransferase family, *Dnmt1*, *Dnmt3a*, and *Dnmt3b*; Group C contains markers of primordial germ cells such as *Blimp1*, *Fragilis*, *Stella*, *cKit*, *Pumilio1* (*Pum1*), *Pumilio2* (*Pum2*), and *Nanos3*; Group D contains the gonocyte markers *Vasa*, *Stra8*, *Sycp3 (Synaptonemal Complex Component 3)*, *GCNF*, and *GDF9 (Growth Differentiation Factor 9)*; Group E contains the late germ cell markers *Tdrd1* (*Tudor domain containing 1*), *Tekt1* (*tektin1*), and *Acrosin*; Group F contains pro-apoptotic markers *Casp6* (*Caspase6*) and *Bax* (*BCL2-associated X*), as well as the anti-apoptotic marker *Bcl*-2; Group G contains markers of autophagy including *Atg5* (*autophagy related* 5) and *Beclin1*.

Male *Oct4*ΔPE:GFP-positive cells isolated from wildtype and *Dazl* heterozygous lines had significant expression of most genes from the pluripotency and germ cell specific groups (A–D) during days 0–7, followed by down-regulation in subsequent days of differentiation. However, while *Dazl*-null lines expressed pluripotency markers on days 0–7, expression of most epigenetic regulators and germ cell specific genes (groups B–D) was greatly reduced relative to wildtype and heterozygous lines. Remarkably, this was observed even in the undifferentiated state. Interestingly, *Dazl* heterozygous cells show a high level of the late germ cells marker (Group E) relative to both wildtype and *Dazl*-null cells during days 0–7; however, all genotypes appeared to maintain low levels of groups A–E genes on days 14–35. Furthermore, wildtype male lines maintained relatively low levels of the cell death markers (Groups F & G) throughout differentiation, whereas *Dazl* heterozygous and *Dazl-null* lines expressed high levels of the pro-apoptotic gene *Bax* (group F) and the autophagy marker *Atg5* (group G) on days 0–7 (Figure 7). These results suggest that the male wildtype and *Dazl* heterozygous lines are undergoing an initial wave of differentiation towards the germ cell lineage, which is reduced in the *Dazl* null lines, and that relative to wildtype, male cells with *Dazl* ablation may have a slightly higher level of cell death.

**Figure 8:**
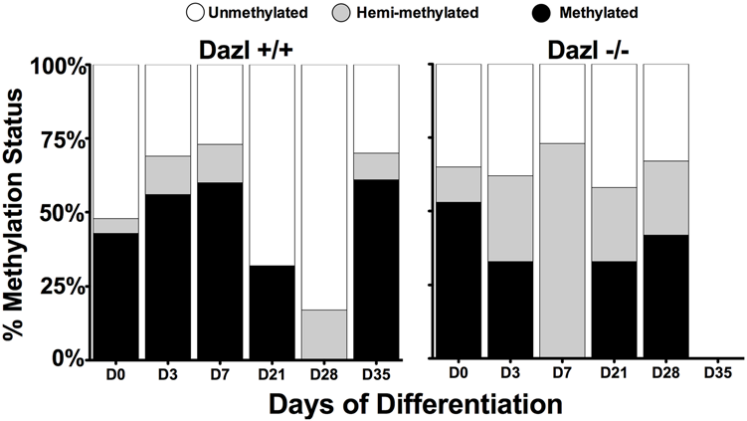
*Dazl* is required for Erasure and Re-establishment of H19 DMR Imprints. Results of bisulphite sequencing on GFP-positive germ cells isolated by FACS, displaying an average percent methylation status (y-axis) and days of differentiation on the x-axis. Average values are from 40 clones per datapoint (20 clones per individual sample, 2 replicates for each genotype/timepoint).

Female *Oct4*ΔPE:GFP-positive cells from wildtype and *Dazl* heterozygous mESC lines displayed high levels of expression of the pluripotency associated genes (Group A) on days 0–14, while epigenetic regulators, PGC markers, and later gonocyte markers (Groups B–D) showed high expression on days 0–7. Furthermore, Group D expression continued through to day 21 of differentiation, particularly in wildtype lines. Markers of apoptosis and autophagy (Groups F & G) also showed relatively high expression early in the time course until day 14 in wildtype and heterozygous lines. Notably, wildtype and *Dazl* heterozygote cells down-regulated most of the genes in groups A–G by days 21 and 28, with a second wave of expression ensuing by day 35, albeit, this was much more striking in the wildtype lines. In contrast, we observed that *Oct4*ΔPE:GFP-positive cells isolated from *Dazl-null* samples showed dramatic differences in gene expression relative to wildtype and heterozygous lines, even on day 0, which continued across all timepoints, and encompassed most of the expression groups (Figure 8A–E). We conclude that as in the male lines, wildtype and *Dazl* heterozygous female lines displayed an initial wave of germ cell development through day 14 of differentiation, followed by a second wave at day 35, seen more strongly in wildtype samples. We note that similar to *Dazl* null male lines, the female line lacking *Dazl* showed an elevation of apoptotic and autophagy markers around day 7, however, the other female genotypes displayed similar expression of these markers at this timepoint, therefore it is difficult to suggest the lines lacking *Dazl* are undergoing higher levels of cell death. Furthermore, our results in female lines suggest that in the absence of *Dazl* differentiation towards the germ cell lineage is impaired as early as day 0 and persists throughout differentiation.

### *Dazl* and H19 DMR Methylation *in vitro*

To determine whether the correct cycle of germ cell imprint erasure and re-establishment occurs *in vitro* in the absence of *Dazl*, we examined methylation at the H19 DMR in germ cells differentiated from male wildtype and *Dazl*-null mESC lines. Results indicated that at day 0, wildtype and *Dazl−/−* null mutant lines have an approximately 50% methylated, 50% unmethylated pattern, as is expected in undifferentiated mESCs (Figure 8, day 0). Interestingly, data for day 3 and 7 show that the wildtype lines remain close to the 50:50 pattern seen in undifferentiated cells, while the *Dazl* null cells appear to be undergoing demethylation, however, most of the clones from the *Dazl* null samples are not completely unmethylated, but instead 73% are hemi-methylated. Furthermore, by day 21, the wildtype lines are increasingly unmethylated, until by day 28, 83% of clones are unmethylated, and the remaining 17% are only hemi-methylated and by day 35 wildtype lines have initiated re-establishment of methylation with 61% of clones fully methylated. Conversely, GFP positive germ cells from *Dazl−/−* mutant lines, despite the decrease in methylation seen at day 7, never fully erase the methylation marks. Instead, at day 21 they display a similar percentage of methylated, unmethylated, and hemi-methylated clones as seen at day 0, and maintain this status until day 28. Unfortunately, the small number of GFP-positive cells collected from *Dazl* null lines at day 35 negated ability to analyze epigenetic modifications at this time point.

## Discussion

Several studies have examined phenotypes associated with disruption of germ cell specific genes, such as *Dazl*, in mice but most rely on histochemical and whole tissue analysis as purification and analysis of small numbers of germ cells in early development has been difficult [20], [22], [26], [52]. In this study, we used the *Oct4*ΔPE:GFP transgene [40] to isolate small numbers of germ cells *in vivo* and *in vitro*. We demonstrate that ablation of a single translational factor, *Dazl*, disrupts a continuum of fundamental genetic and epigenetic events of post-migratory germ cell development both *in vivo* and *in vitro*. Furthermore, although previous work has documented the need for the transcriptional regulator *Prdm14* in specification of germ cell fate and commitment to the reprogramming events necessary for appropriate early germ cell development [51], our data is focused on the translational (not transcriptional) factor, *Dazl*. Moreover, we show that ablation of *Dazl* results in a reduction in post-migratory germ cell numbers, aberrant expression of markers of pluripotency and differentiation, failure to execute nuclear reprogramming, failure to produce EG lines and inability to progress through meiosis, even in the presence of *Prdm14* (Figure S6), suggesting that *Dazl* is genetically downstream of *Prdm14* or functions in a parallel pathway.

In this report we show a quantitative and significant decrease of germ cell numbers *in vivo*, independent of sex, by E14.5 in *Dazl−/−* mutant gonads relative to wildtype. This is in contrast to earlier characterizations of the mouse *Dazl*−/− mutant, which suggested that germ cell loss occurs only in males at E14.5 [52], or begins later in embryonic development at approximately E15.5 to E17.5 [22]. As our results are described on a genetic background similar to the one reported by Ruggiu et al [22], (with the addition of the FVB background from the *Oct4*ΔPE:GFP transgenic mice [40]), we suggest that the difference in these results may be due to the our use of FACS to capture and characterize PGCs, thus enabling us to uncover much earlier quantitative and qualitative defects in *Dazl* null PGCs. Furthermore, our results in males at E14.5 are comparable to those found by Lin et al. after they performed C57BL/6 backcrossing to the *Dazl* mutant mice used here [26], although the same group did not uncover similar defects in PGC number in *Dazl* null females [52]. This difference may be due to an increased variability, or potential differences in flanking regions, often described in mixed genetic backgrounds [53]–[54], however, we can not discount that differences could also arise from the increased sensitivity the use of FACS provides when capturing PGC data in females at these embryonic timepoints. Here we report that regardless of whether the germ cell programs mandate entry into meiosis for female germ cells or mitotic arrest in male germ cells, germ cell numbers are diminished in the absence of *Dazl* function. Indeed, in spite of intra-genotype variation, we detected statistically significant differences in germ cell numbers early in development with ablation of *Dazl* by E14.5. The differences in the numbers of germ cells amongst mice of different genotypes are magnified further by E16.5. We also note that by quantification we observed a more severe reduction in germ cell number at E15.5 and E16.5 in male *Dazl* mutants than in females, suggesting a sex-specific difference in the kinetics of germ cell loss.

Results of quantitative analysis were mirrored by studies of gene expression. Beginning at E13.5, there were observable and reproducible differences in the expression of certain genes in wildtype versus *Dazl−/−* germ cells. *Dazl−/−* male germ cells had relatively low expression of early germ cell markers such as *Pumilio-2*, *Stella* and *Oct-4*, while mutant female germ cells had relatively high expression of these same markers relative to their wildtype littermates. We also note that the striking differences in *Stella* expression between wildtype male and female PGCs occurring at E14.5 is consistent with previous reports [55]. Markers that normally begin to be expressed after germ cells reach the gonad, such as *Vasa*, *SYCP3*, and *SYCP1*, showed reduced expression in *Dazl−/−*mutants of both sexes. As the majority of migrating germ cells should have arrived at the gonad by E12.5, this data suggests that loss of *Dazl* function may result in defects in germ cell proliferation, differentiation and/or maintenance with substantial germ cell loss manifested in the gonocyte population of both sexes.

Additionally, we explored other hallmarks of *in vivo* germ cell development, including imprinting and entry into meiosis. We found that *Dazl*−/− males displayed a delay or incomplete erasure at E13.5, and failure to properly re-establish methylation of genomic imprint marks at the H19 DMR, relative to wildtype and heterozygous animals. We note that it is possible that disruption of *Dazl* may directly or indirectly impact imprinting status; previously, germ cell specific genes, other than components of the cellular enzymatic machinery such as the demethyltransferase family, have not been shown to impact germ cell imprinting. Furthermore, we observed that many of the PGCs isolated from *Dazl*-null mutants were able to enter meiosis, however these cells displayed a significant defect in progression through meiosis relative to wildtype. This is in contrast to results obtained with whole gonad gene expression and immunostaining suggesting that loss of *Dazl* blocks entry in to meiosis [52]. Thus, the use of quantification and *Oct4*ΔPE:GFP to isolate germ cells at multiple timepoints allows uncovering of novel phenotypes relative to other methods [22], [26], [52].

**Figure 9:**
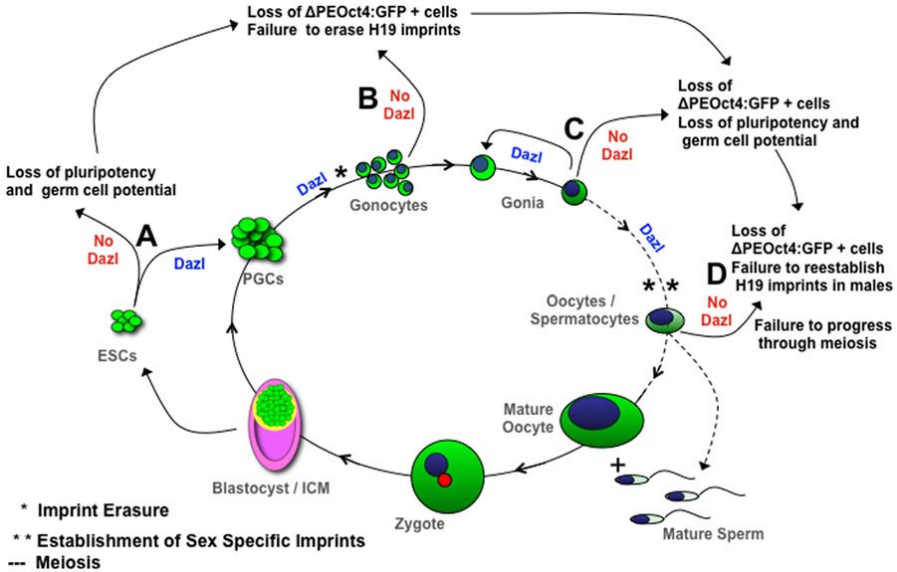
Proposed Model Denotes Dazl as Link Between Pluripotency, Reprogramming and Differentiation *in vivo* and *in vitro*. Green signifies Oct4ΔPE:GFP expression during the lifecycle of germ cell development. (A) mESCs derived from *Dazl*−/− blastocysts display a reduction in pluripotency and germ cell markers, and do not differentiate towards the germ cell lineage as seen in wildtype and *Dazl* heterozygous mESC lines. (B) Gonocytes *in vivo* and putative germ cell *in vitro* show a reduction in Oct4ΔPE:GFP -positive cells, and do not display stage specific genetic markers of germ cell development, or erasure of imprinting methylation marks at the H19 locus in males. (C) Gonia isolated from *Dazl*−/− mutant gonads show a significant reduction in Oct4ΔPE:GFP -positive cells and aberrant gene expression, indicating a loss of pluripotency, self-renewal, and germ cell potential. (D) Early stage oocytes and spermatocytes from *Dazl*−/− embryos display a significant loss of Oct4ΔPE:GFP -positive cells, aberrant gene expression, failure to re-establish imprinting methylation marks at the H19 locus in males, and failure progress though meiosis.

One of the critical links in establishing a robust genetic system for *in vitro* analysis of gene function has been the absence of data to relate findings to those *in vivo* (outlined in Figure 9). Utilizing the same unique combination of germ cell-specific reporter and *Dazl* knockout, we confirmed *in vitro* that ablation of *Dazl* leads to a reduction in *Oct4*ΔPE:GFP-positive putative germ cells during differentiation from mESCs. Interestingly, the use of this system also uncovered a more pronounced defect in *Dazl* heterozygous ESCs than seen in *Dazl* heterozygous PGCs *in vivo*, where *Dazl* heterozygous embryos had a non-significant reduction in germ cell number and little to no change in gene expression or H19 imprinted status relative to wildtype. Our finding *in vitro*, that *Dazl* heterozygous cell lines display a similar defect in GFP-positive cell number as seen in homozygous mutants, suggests that in the absence of gonadal somatic support, one copy of *Dazl* may not be sufficient to support germ cell development *in vitro*, however further work is needed to determine the validity of this hypothesis.

Furthermore, as seen in other reports [28]–[34], our wildtype ESC-derived germ cells show elevated expression of early and late germ cell markers and display appropriate erasure and re-establishment of imprinted methylation at the H19 locus. The high expression of pluripotency, epigenetic regulator, and early and late germ cell-associated genes at early time points of differentiation in both male and female wildtype and *Dazl* heterozygous lines suggests that mESC lines that contain at least one copy of *Dazl* are able to undergo an initial wave of germ cell differentiation. However, only the female wildtype lines displayed a strong second wave of germ cell differentiation at day 35. We note that the *Oct4*ΔPE:GFP transgene has been reported to decrease in expression in germ cells undergoing spermatogenesis (39), therefore, lack of induction of germ cell markers at later stages in male wildtype lines could be due to loss of maturing germ cells as they down-regulate *Oct4*. Analysis of the Oct4ΔPE:GFP-negative fraction from male wildtype lines at day 35 supports this suggestion (data not shown). Furthermore, we did not see these results in ESCs with a genetic ablation of *Dazl*. In these lines, in particular in the female line, the drastic reduction in expression of pluripotency and epigenetic regulating genes, as well as the early and late germ cells markers, as early as day 0, and persisting throughout all timepoints of differentiation, suggests that *Dazl* is linked to germ cell potential even at early fate decisions of *in vitro* differentiation, and supports the conclusion that *Dazl* is critical to maintaining a population of mESCs capable of germ cell fate.

As illustrated in our model (Figure 9), we suggest that *Dazl* functions early *in vivo* or in differentiating mESCs *in vitro*, either directly or indirectly, to maintain germ cell pluripotency. We further suggest that this RNA-binding protein may function to translationally-regulate or interact with key factors required to guide the germ cells through development, from stage to stage, and is required for the post-migratory pluripotent germ cells to begin reprogramming and switch from mitotic to meiotic divisions (Figure 9A–9D).

We also wish to emphasis two items of interest arising from our *in vitro* results. The first addresses a common and perplexing problem of *in vitro* differentiation of ESCs, namely the persistence of a residual *Oct4*-positive population that is thought to be tumorigenic in transplant assays following somatic differentiation [56]–[60]. Here we demonstrate that the use of ESCs carrying a disruption of a germ cell-specific gene, *Dazl*, significantly reduced or abolished the *Oct4* residual population. Even when retinoic acid, shown to promote self-renewal of ESCs [61] is added to the media, we still see a complete reduction of GFP-positive cells in *Dazl* null cell lines (data not shown). Thus, our results suggest that the residual *Oct4* population of cells is not a population of ESCs resistant to differentiation but instead is the population of cells that has differentiated to the germ cell lineage rather than the somatic lineages. This implies that all differentiation of ESCs may be a balance between somatic and germ cell fates.

Finally, we note that this is the first demonstration that a genetic ablation leads to failure to produce germ cells *in vitro* with altered gene expression beginning at Day 0 in undifferentiated ESCs. This observation supports the hypothesis that a *Dazl*-dependent subpopulation of cells in undifferentiated ESC colonies may be predisposed towards differentiation to the germ cell lineage (Figure 9A). Interestingly, a recent report documented evidence of heterogeneity within undifferentiated mESC colonies based on presence or absence of *Stella*, which leads to differences in differentiation potential [62]. Unlike our results, this work reported a dynamic equilibrium between the *Stella*-positive and *Stella*-negative populations [62], whereas in our system, genetic ablation of *Dazl* appears to irreversibly preclude mESC differentiation to the germ cell lineage and/or maintenance of pluripotent germ cell identity (Figure 9A–9D). Further analysis utilizing this system will help uncover how the fundamental decision between formation and maintenance of the *Oct4*-residual population and differentiation to the germ cell lineage is coordinated by a gene that encodes an RNA-binding translational regulator. In conclusion, the data presented in this report shows that *Dazl* serves as a molecular linker between maintaining the pluripotent germ cell population and execution of the epigenetic and genetic programs that define multiple time points of germ cell differentiation *in vitro* and *in vivo*.

## Materials and Methods

### Mice

The Oct4ΔPE:GFP transgene [36]–[40] and *Dazl* null allele [22] have previously been reported. Mice carrying the Oct4ΔPE:GFP reporter transgene [40] were mated to *Dazl+/−* mice to produce mice that were heterozygous for both the reporter and the *Dazl-null* allele. Male and female double heterozygotes were then mated to produce progeny for analysis. For mESC derivation, females were superovulated 48 hours prior to breeding by injection with 5 units pregnant mare serum gonadotropin (PMSG, Sigma-Aldrich). Immediately before placing the female with a male they were injected with 5 units (suspended in 100 µl PBS) human chorionic gonadotropin to hyperstimulate ovulation. The morning that the copulation plug was observed was considered 0.5 days post coitum (dpc). On different embryonic days, pregnant females were sacrificed and blastocysts or embryos removed from the uterus and placed in PBS for analysis or onto a feeder layer for mESC line derivation. Embryonic somatic tissues or feeder-free ESCs were used to genotype for *Dazl*, Oct4ΔPE-GFP, and *Sry* for sexing as previously reported [22], [39], [63]. Care and use of experimental animals described in this work comply with guidelines and policies of the Administrative Panel on Laboratory Animal Care at Stanford University.

### Mouse Embryonic Stem Cell Derivation, Maintenance, and Differentiation

Matings of male and female double heterozygotes were set up as above. At 3.5 dpc females were sacrificed and uterine horns were excised placed in mESC media (DMEH21 (Gibco)+10% FBS (Hyclone), 1× pen-strep (UCSF Cell Culture Facility), NEAA (Gibco), BME (Sigma), and LIF (Chemicon)). Blastocysts were flushed from uterine horns and transferred into 100 ul of fresh media repeatedly until maternal blood was completely removed. Whole blastocysts with intact zona pellucida were plated into one well of a 6-well plate, containing irradiated mouse embryonic fibroblasts (feeder layer) plated 24 hours earlier, along with fresh mESC media. Blastocysts were incubated at 37°C in 5% CO2 for 7–14 days, until visible outgrowths were observed. Outgrowths were detached and transferred individually to one well of a 96-well plate containing 30 ul of 0.25% Trypsin/0.025 EDTA and left at room temperature for 5–10 minutes until dissociated. Trypsin was neutralized using fresh mESC media and cells were transferred to one well of a 24-well plate onto feeders and incubated as above. Once expansion was obtained, cells were cryo-preserved and used in differentiation experiments. For genotyping, each line was also passaged onto 0.01% gelatin and passaged repeatedly to provide feeder-free mESCs. We performed metaphase spreads to karyotype our mESC lines as follows. Undifferentiated mESCs were treated for 1 hour with KaryoMax colcemid treatment (10 uL/mL; Invitrogen), followed by 7 minutes in hypotonic solution (1∶1; 0.4% KCl∶0.4% Sodium Citrate), at 37°C, followed by a 50-minute fixation in 3∶1, methanol to acetic acid solution. We then dropped the cells onto glass slides (Fischer Scientific) and after air-drying used a 4% Giemsa/Gurr buffer solution to stain the chromosomes. We used a Lecia DFC300FX R2 camera, to visualize the spreads, and a minimum of 20 individual cells per line were selected for karyotypic analyzed. Undifferentiated mESC lines were maintained and expanded in mESC media on feeder layers. To differentiate the mESCs we used a suspension protocol described previously [34]. In brief, following enzymatic dissociation using 0.05% trypsin (Invitrogen) to single cell, we placed ∼250,000 cells/well in 3 mL differentiation media (mESC media with 20% FBS, minus LIF) in low adhesion 6-well plates (Corning).

### Embryonic Gonad and Embryoid Body Dissociation

Gonads were dissected from embryos and placed in DMEM/F-12 medium with 20% fetal bovine serum (DMEM/F-12+FBS), while EBs were harvested from 6 well plates. Both were pelleted at 2000 rpm for 3 min and the supernatant was removed. The samples were then dissociated enzymatically with 1 mg/ml of collagenase/dispase (Roche Molecular Biochemicals) in PBS for 30 min at 37°C, and pelleted. Media was replaced with PBS containing 0.25% trypsin, 0.2% Versene. Samples were incubated 20 min at 37°C and then treated with DNase I (Roche Molecular Biochemicals) at a final concentration of 1 mg/ml for 10 min at 37°C. Cells were dissociated to a single-cell suspension by pipetting and DMEM/F-12+FBS was added to inactivate trypsin. Finally, cells were pelleted, washed with DMEM/F-12+FBS or differentiation medium, and resuspended in 0.5 ml media for analysis.

### Embryonic Germ Cell Derivation

Gonads from E12.5, E14.5, E15.5 and E16.5 wildtype, *Dazl* heterozygote, and *Dazl* null embryos were isolated, genotyped, and dissociated as above. Once sex and genotype were confirmed we combined multiple samples of the same sex/genotype combination and then isolated Oct4ΔPE:GFP-postivie germ cells by FACS. After sorting we plated the purified cell on previously plated STO-feeders in PGC defined media that included LIF, SCF, and Fgf2 following the protocol described by De Miguel and Donovan [50]. After plating daily observations were taken on a Leica DM IL HC Leica to determine morphology, GFP expression, and colony formation in each well. Images were acquired with a Lecia DFC300FX R2 camera.

### Fluorescence-Activated Cell Sorting (FACS)

For FACS, dissociated gonadal cells were resuspended in PBS+1% bovine serum albumin (BSA) with propidium iodide (PI; Roche Molecular Biochemicals) at a final concentration of 10% to mark necrotic cells and analyzed on a CyAn ADP analyzer (Dako). GFP-positive cells were counted; a negative control (no GFP transgene) was included in all experiments. To collect germ cells for later analysis, cells were prepared similarly as for counting, but collected on a MoFlo High-Performance Cell Sorter (Dako), then stored at −80°C until use. Dissociated EBs were resuspended in differentiation media with 0.25 µg per sample of 7-Amino-Actinomycin D (BD Biosciences) or PI to exclude nonviable cells. The EB suspension was analyzed and GFP-positive and GFP-negative cells were collected on the FACSAria Cell-Sorting System (BD Biosystems).

To analyze cells for DNA content, dissociated germ cells were fixed overnight at 4°C in 4% paraformaldehyde. The next day, cells were permeabilized by resuspension in 0.2% Triton X-100 in PBS for 15 min at room temperature. Cells were then washed with cold PBS+1% BSA, and resuspended in PBS with 1 unit of DNase-free RNase (Roche Molecular Biochemicals) for 30 min at 37°C. Finally, 300 ul of PBS+1% BSA and 40 ul of PI were added and cells were analyzed on a FACSCalibur analyzer (BD Biosciences).

### Microscopy

To examine nuclear morphology of the germ cells, dissociated germ cells were fixed, permeabilized, and washed as described for analysis of DNA content. Cells were then resuspended in 100 µl of DAPI for 15 min at room temperature. After staining, the cells were resuspended in 15 µl of cold PBS+1% BSA and applied drop-wise to a poly-L-lysine slide. Slides were dried and sealed with Prolong Antifade Gold (Invitrogen). Cells were visualized by immunofluorescence. To examine meiotic competence we isolated Oct4ΔPE:GFP-positive cells and performed meiotic spreads using a modified version of Gonsalves et al., [64], followed by immunofluorescence staining using rabbit polyclonal SYCP3 antibody (Abeam) to label aligned chromosomes, and Prolong Antifade Gold (Invitrogen) to label the nucleus.

### RNA Extraction, cDNA Construction, and Real Time PCR

For *in vivo* samples RNA was extracted from previously sorted germ cells and/or EBs using the Picopure RNA Extraction kit (Arcturus Bioscience) as previously described [65]. cDNA was then constructed, according to manufacturer's protocol, using either the iScribe cDNA Synthesis (BioRad Laboratories) or SuperScript II (Invitrogen) systems. For real time PCR, each reaction contained 2 µl cDNA, 1 µl of each primer, 10 µl of iQ SYBR Green Supermix (BioRad), and 6 µl water. The reactions were run and analyzed on a MyIQ PCR system (BioRad) with the following protocol: 95°C×5 min; 45 cycles of 95°C×30 sec, 60°C×1 min, 72°C×1 min; 72°C×5 min. All reactions were also checked by gel electrophoresis for the presence of primer dimers. The expression of each gene was normalized to *GAPDH* expression using the following formula: 2^-(gene CT value – GAPDH CT value)^. The efficiency of GAPDH was tested at multiple cDNA concentrations and all real-time curves were evaluated post-run to ensure each sample had similar primer efficiencies. Furthermore, GAPDH maintained a steady expression across all developmental timepoints tested. Primers were as indicated (Table S1).

For *in vitro*-derived samples, we used the BioMark Dynamic Array (Fluidigm Corporation) microfluidics system for RT PCR. We pre-amplified samples by treating 50 GFP-positive cells per mESC line per time point following manufacturer's protocol (Fluidigm Corporation) using 20× Taqman gene expression assays (Applied Biosystems; Table S2) as specified in Figure 8 and Table S2. For samples, the reaction mix contained 2.5 uL 2× Universal Master Mix (Applied Biosystems), 0.25 uL Sample Loading Buffer (Fluidigm Corporation), and 2.25 uL pre-amplified cDNA for loading into the sample inlets of the 48 by 48 Dynamic Array (DA)(Fluidigm Corporation). For probes, the reaction mix contained 2.5 uL 20× Taqman Gene Assay and 2.5 uL Assay Loading Buffer (Fluidigm Corporation) for loading into the assay inlets on the DA. Each sample had 2 technical replicates and average CT values were calculated, and normalized to GAPDH. Analysis included samples for each mESC line from at least 2 independent experiments, for all time points, and combined results from mESC lines of similar sex/genotype combinations. Results are displayed on DChip (http://www.dchip.org/) in heat map format.

### H19 Imprinting Analysis

Genomic DNA was isolated from FACS purified GFP-positive cells using the ZR Genomic DNA II Kit (Zymo Research) and concentration was assessed using the NanoDrop ND-1000 UV-Vis Spectrophotometer (Nanodrop Technologies). 500 ng of gDNA (or total sample if less than 500 ng) per sample was used for bisulfite conversion and subsequent sequencing. Bisulfite conversion was performed using the EZ DNA Methylation Kit (Zymo Research) or EpiTect Bisulfite Kit (Qiagen). 2.5 µl of bisulfite-treated gDNA was used as a template to amplify region 1309–1726 of the H19 differentially methylated region (DMR) containing 15 CpG sites (GenBank acc. No. U19619) as described previously [66]–[67]. PCR product was subcloned using TOPO TA Cloning Kit pCR®2.1 (Invitrogen) and 20 colonies per sample were sequenced. Samples with less than 95% bisulfite conversion of cytosine to thymine in non-CpG cytosine positions, and/or incomplete sequences were not considered in the analysis. To determine whether methylation had been erased in male germ cells, germ cells were collected from gonads of E13.5 embryos. To determine if imprints had been re-established correctly, germ cells were collected from gonads of E16.5 embryos and 1-day post partum neonates. Wildtype, heterozygous, and homozygous *Dazl-null* individuals were assayed; heart tissue was used as a somatic control. For each genotype/age group in the study, analysis encompassed three individuals and for each unique sample, 20 clones. The methylation status at 15 CpG sites was scored and each clone was assigned a status of methylated (at least 75% of sites methylated), unmethylated (at least 75% of sites unmethylated), or hemi-methylated (approximately 50% methylated, 50% unmethylated). Results were averaged across samples in the same genotype/age group. Samples collected on days 0–35 of *in vitro* differentiation from mESC lines were analyzed as above, from wildtype and *Dazl−/−* mutant lines.

### Statistics

Data for germ cell counts were fit to a negative binomial regression model using Stata v6.0 (Stata Corporation). Error bars for gene expression were calculated in Excel using the standard deviation function. Data for mESC differentiation experiments and H19 imprinting analysis were analyzed and graphed on Prism 4.0c for Macintosh (GraphPad Software, Inc.) using 2 way ANOVA with Bonferroni post-tests.

## Supporting Information

Figure S1 Gene Expression During Germ Line Stem Cell Development. Diagram of germ line development from specification to sex-specific differentiation (sperm or egg, modified from Clark et al., 2004). In the lower half of the figure, expected gene expression for the given stage of development is displayed. Selected genes include Oct4 (Octamer 4), Blimp1, Nanog, Stella, Pum2 (Pumilio 2), Nanos, TNAP (Tissue-Nonspecific Alkaline Phosphatase), c-Kit, Dazl (Deleted in Azoospermia-Like), Vasa, Sycp1 (Synaptonemal Complex Protein) 1 and 3, Tekt1 (tektin1), a marker for adult spermatids, and GDF9 (Growth and Differentiation Factor 9), a marker for adult oocytes. ‘*’ denotes the point in development as when the primordial germ cells begin to erase imprinting methylation marks from their DNA. ‘**’ denotes the stage in development when germ line stem cells diverge and begin establish a sex-specific imprinting pattern of DNA methylation.
(9.75 MB TIF)

Figure S2 GFP Expression from Oct4ΔPE Promoter is Restricted to Germ cells and Reduced in Dazl-null Gonads. (A) Whole mount from E14.5 wildtype embryonic testis. (B) Whole mount from E14.5 wildtype embryonic ovary. (C) Whole mount from E14.5 Dazl-null embryonic testis. (D) Whole mount from E14.5 Dazl-null embryonic ovary. (E) RT PCR analysis of FACS-isolated GFP-positive and GFP-negative cells from E14.5 gonads.
(9.24 MB DOC)

Figure S3 Loss of Dazl Does not Affect Proliferation in Embryonic Germ Cells of Dazl Mutants. Percentage of germ cells, at E13.5, in the G1 and G2/M stages of the cell cycle. Each bar indicates germ cell percentage for one embryo. Within each sex, the same embryos are depicted in both the G1 and G2/M graphs, although not necessarily in the same order within the genotype. The average percentage for each genotype is shown above the bars in parentheses.
(4.66 MB TIF)

Figure S4 GFP Expression from Oct4ΔPE Promoter in mESC lines Becomes Restricted During in vitro Differentiation. Bright field (A, E) and Oct4ΔPE:GFP expression (B, F) in undifferentiated Dazl+/+ and −/− mESC colonies plated on mouse embryonic fibroblasts. Bright field (C, G) and Oct4ΔPE:GFP expression (D, H) in D14 Dazl+/+ and −/− embryoid bodies. The Dazl−/− D14 embryoid bodies display a reduction in GFP-positive foci (H).
(10.01 MB TIF)

Figure S5 Dazl Heterozygote Breeding for mESC Derivation and in vivo PGC Experiments Shows Correct Mendilian Genotype Ratios & Appropriate Chromosomal Composition. A) Graph of genotype percentage from 10 Dazl heterozygous crosses used to derive 44 mESC lines. B) Graph of genotype percentages from 45 Dazl heterozygous crosses used to examine 293 embryos at different stages of embryonic development. C) Representative images of Giemsa stained metaphase spreads of female wildtype, Dazl heterozygous, and Dazl null mESCs.
(6.60 MB DOC)

Figure S6 Prmt14 expression n E12.5 wildtype, Dazl heterozygous, and Dazl null PGCs. RT PCR on Prmt14, Prmt5, and Blimp1 confirms variation but no significant differences (p>0.5) between female (F) and male (M) E12.5 wildtype (wt), Dazl heterozygous (het), and Dazl null (ko)
(9.18 MB TIF)

Table S1 Primers used for in vivo real time PCR
(0.05 MB DOC)

Table S2 Primers used for in vitro real time PCR
(0.06 MB DOC)
