# Supplementary figures and images for: Dazl Functions in Maintenance of Pluripotency and Genetic and Epigenetic Programs of Differentiation in Mouse Primordial Germ Cells In Vivo and In Vitro
Source: PLoS One. 2009 May 21;4(5):e5654. doi: 10.1371/journal.pone.0005654 (PMC2681483; doi:10.1371/journal.pone.0005654)

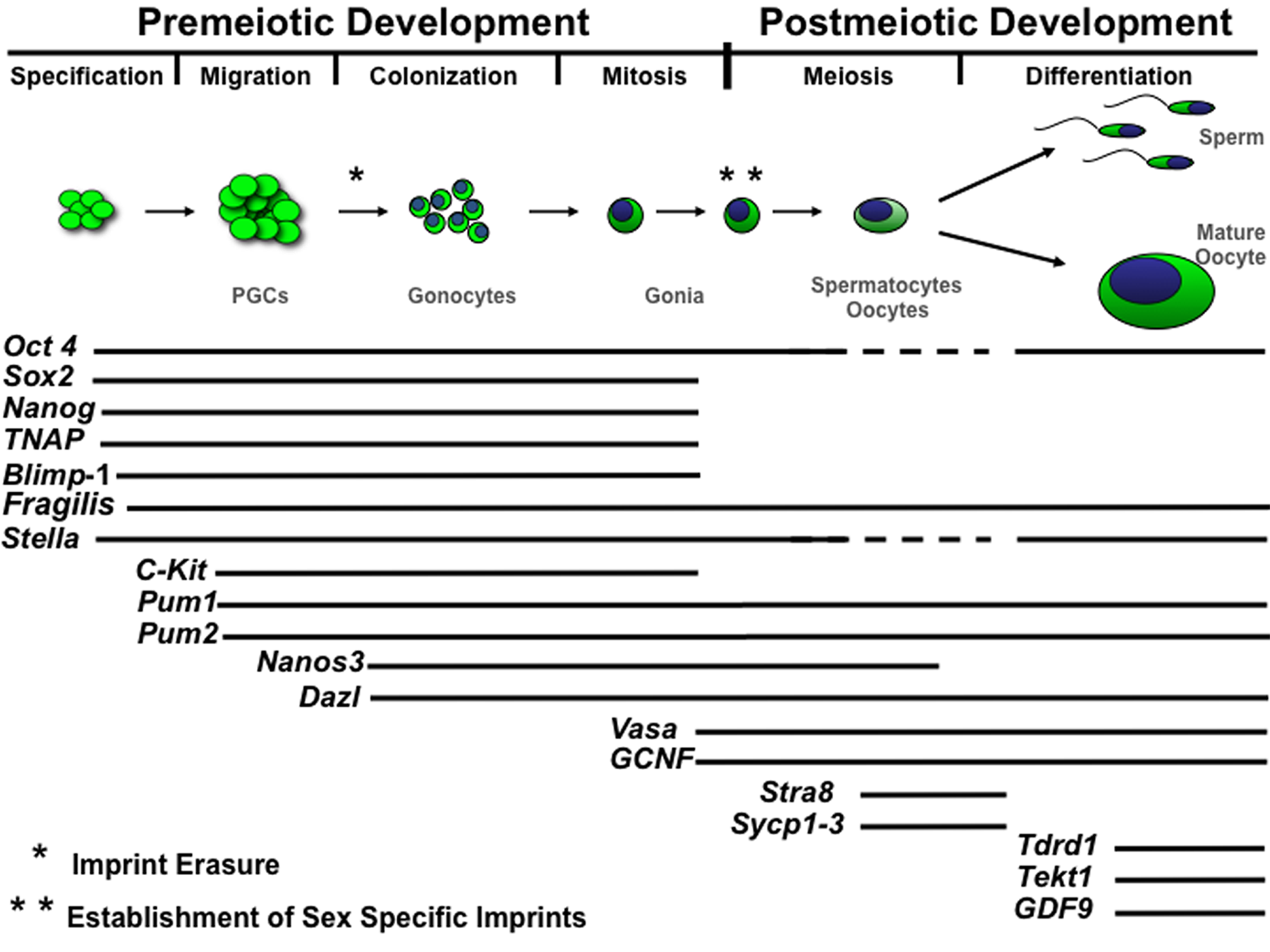

Supplement: Figure S1 — Gene Expression During Germ Line Stem Cell Development. Diagram of germ line development from specification to sex-specific differentiation (sperm or egg, modified from Clark et al., 2004). In the lower half of the figure, expected gene expression for the given stage of development is displayed. Selected genes include Oct4 (Octamer 4), Blimp1, Nanog, Stella, Pum2 (Pumilio 2), Nanos, TNAP (Tissue-Nonspecific Alkaline Phosphatase), c-Kit, Dazl (Deleted in Azoospermia-Like), Vasa, Sycp1 (Synaptonemal Complex Protein) 1 and 3, Tekt1 (tektin1), a marker for adult spermatids, and GDF9 (Growth and Differentiation Factor 9), a marker for adult oocytes. ‘*’ denotes the point in development as when the primordial germ cells begin to erase imprinting methylation marks from their DNA. ‘**’ denotes the stage in development when germ line stem cells diverge and begin establish a sex-specific imprinting pattern of DNA methylation. (9.75 MB TIF) [file pone.0005654.s001.tif]

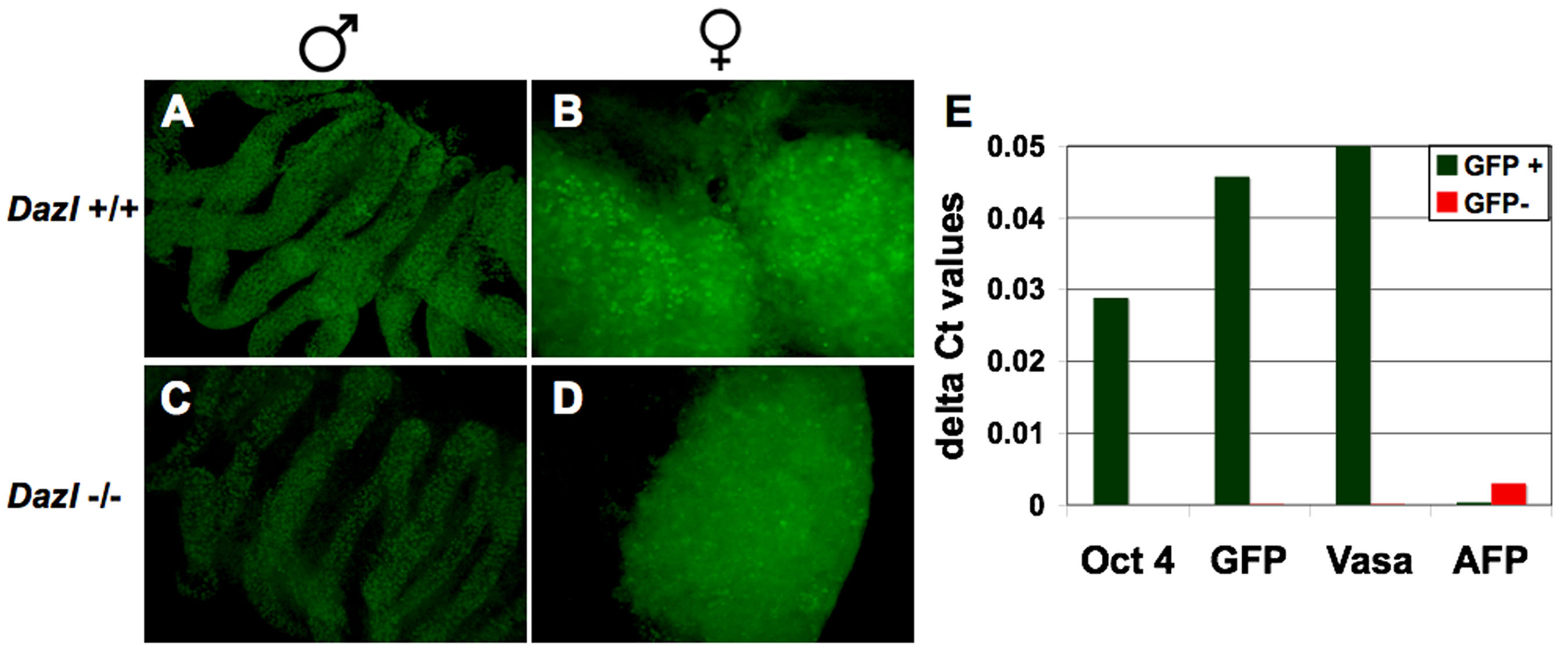

Supplement: Figure S2 — GFP Expression from Oct4ΔPE Promoter is Restricted to Germ cells and Reduced in Dazl-null Gonads. (A) Whole mount from E14.5 wildtype embryonic testis. (B) Whole mount from E14.5 wildtype embryonic ovary. (C) Whole mount from E14.5 Dazl-null embryonic testis. (D) Whole mount from E14.5 Dazl-null embryonic ovary. (E) RT PCR analysis of FACS-isolated GFP-positive and GFP-negative cells from E14.5 gonads. (9.24 MB DOC) [file pone.0005654.s002.tif]

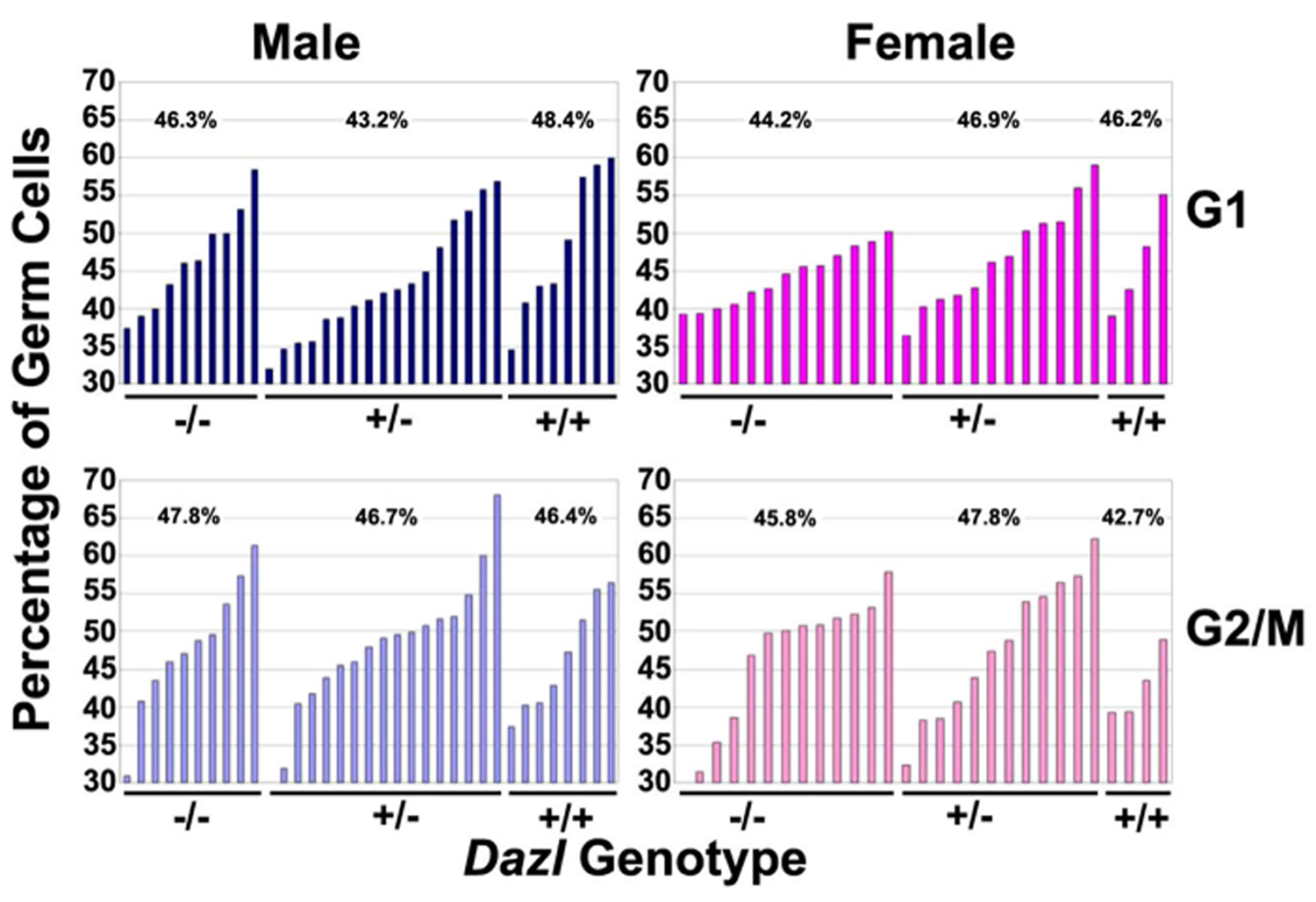

Supplement: Figure S3 — Loss of Dazl Does not Affect Proliferation in Embryonic Germ Cells of Dazl Mutants. Percentage of germ cells, at E13.5, in the G1 and G2/M stages of the cell cycle. Each bar indicates germ cell percentage for one embryo. Within each sex, the same embryos are depicted in both the G1 and G2/M graphs, although not necessarily in the same order within the genotype. The average percentage for each genotype is shown above the bars in parentheses. (4.66 MB TIF) [file pone.0005654.s003.tif]

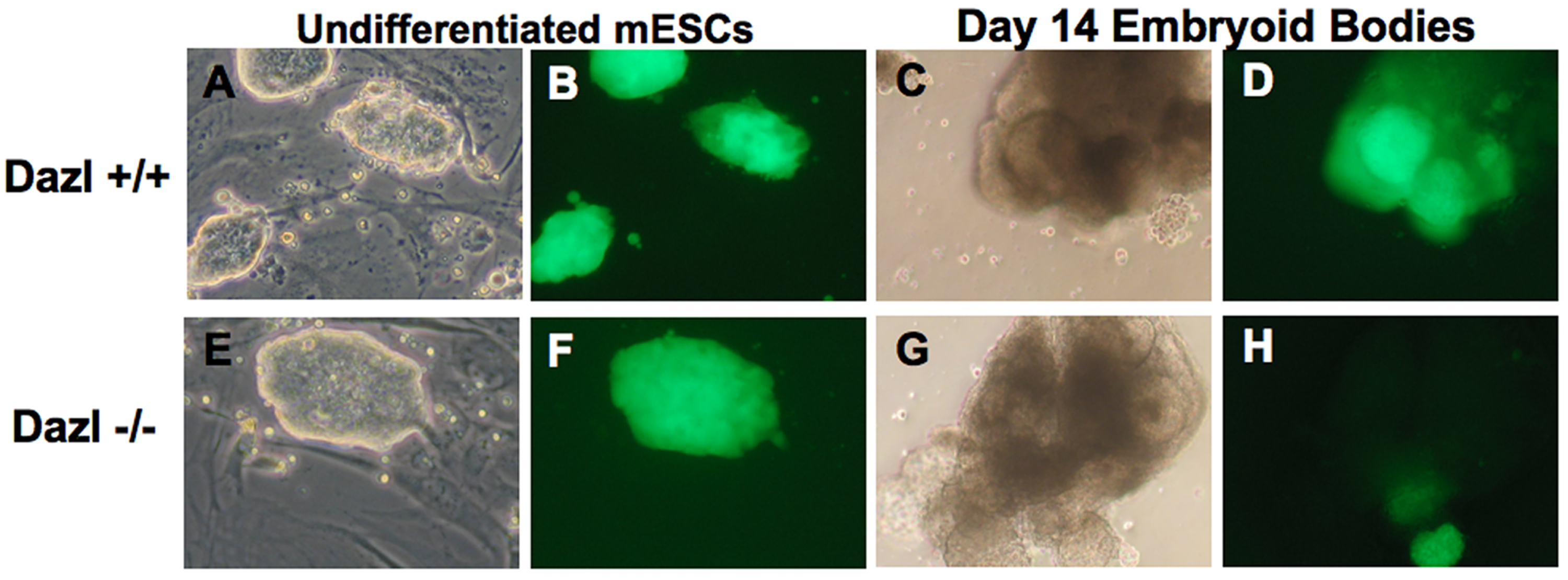

Supplement: Figure S4 — GFP Expression from Oct4ΔPE Promoter in mESC lines Becomes Restricted During in vitro Differentiation. Bright field (A, E) and Oct4ΔPE:GFP expression (B, F) in undifferentiated Dazl+/+ and −/− mESC colonies plated on mouse embryonic fibroblasts. Bright field (C, G) and Oct4ΔPE:GFP expression (D, H) in D14 Dazl+/+ and −/− embryoid bodies. The Dazl−/− D14 embryoid bodies display a reduction in GFP-positive foci (H). (10.01 MB TIF) [file pone.0005654.s004.tif]

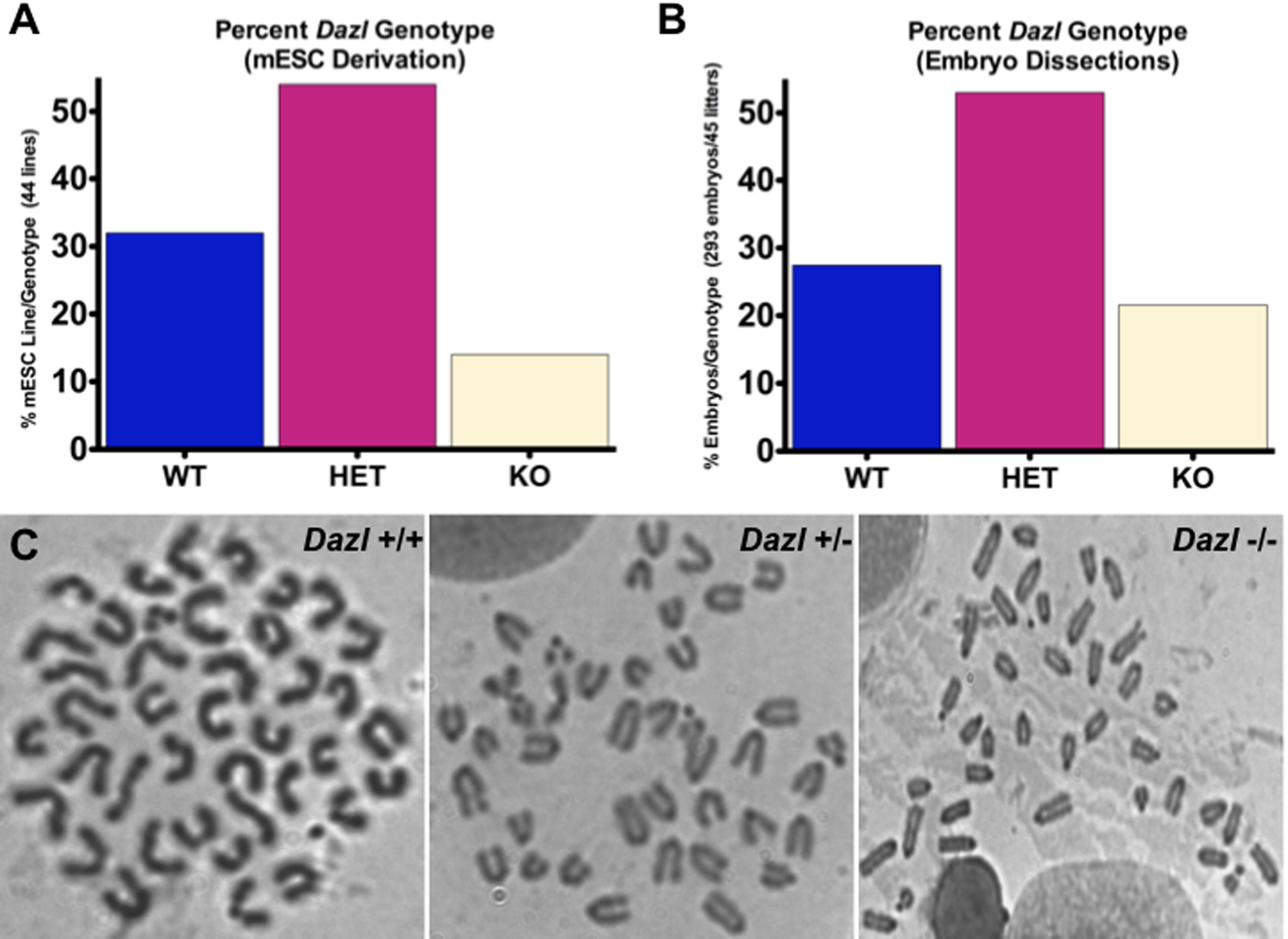

Supplement: Figure S5 — Dazl Heterozygote Breeding for mESC Derivation and in vivo PGC Experiments Shows Correct Mendilian Genotype Ratios & Appropriate Chromosomal Composition. A) Graph of genotype percentage from 10 Dazl heterozygous crosses used to derive 44 mESC lines. B) Graph of genotype percentages from 45 Dazl heterozygous crosses used to examine 293 embryos at different stages of embryonic development. C) Representative images of Giemsa stained metaphase spreads of female wildtype, Dazl heterozygous, and Dazl null mESCs. (6.60 MB DOC) [file pone.0005654.s005.tif]
