# Supplementary material for: Dazl Functions in Maintenance of Pluripotency and Genetic and Epigenetic Programs of Differentiation in Mouse Primordial Germ Cells In Vivo and In Vitro
Source: PLoS One. 2009 May 21;4(5):e5654. doi: 10.1371/journal.pone.0005654 (PMC2681483; doi:10.1371/journal.pone.0005654)
Supplement: Table S1 — Primers used for in vivo real time PCR (0.05 MB DOC) [file pone.0005654.s007.doc]

| **G** | **Sequence** |
| --- | --- |
| *Pumilio2* | Forward: TATCAATTAATGACAGATGTTTTTGGA |
|  | Reverse: CTGATCAGAAGAAATAGATTCTAACGC |
| *Nanos2* | Forward: GGAATAAGAGGAAGGTACAGGAACTA |
|  | Reverse: TATATTGGATGGGTAGAAGAGAGAGAA |
| *Nanos3* | Forward: CTACCTTCGTCTACTGCTACACCAC |
|  | Reverse: ACTTTTGGAACCTGCATAGACACCT |
| *Vasa* | Forward: CTAGGAAGACCAAATAGTGAATCTGAC |
|  | Reverse: TCCAGAACCTGTTACTACTTCTTCATT |
| *Stella* | Forward: CTTTTCAAAGACTAAGCAATCTTGTTC |
|  | Reverse: ATGACCTTTACTAGTGTTTCTGGTTGT |
| *Oct-4* | Forward: AGTCTGGAGACCATGTTTCTGAAGT |
|  | Reverse: TACTCTTCTCGTTGGGAATACTCAATA |
| *c-kit* | Forward: AGAATATTGTTGCTATGGTGATCTTTT |
|  | Reverse: CATGTCCATATATTCATTTGAACTGTC |
| *SYCP3* | Forward: AGAAATGTATACCAAAGCTTCTTTCAA |
|  | Reverse: TTAGATAGTTTTTCTCCTTGTTCCTCA |
| *SYCP1* | Forward: AAGTTTGATTCTAAAACAACTCCTTCA |
|  | Reverse: ACTCTTTTTAGTTGGTGTCTTCACTGT |
| *GAPDH* | Forward: TTCACCACCATGGAGAAGGC |
|  | Reverse: GGCATGGACTGTGGTCATGA |
