# Supplementary material for: Dazl Functions in Maintenance of Pluripotency and Genetic and Epigenetic Programs of Differentiation in Mouse Primordial Germ Cells In Vivo and In Vitro
Source: PLoS One. 2009 May 21;4(5):e5654. doi: 10.1371/journal.pone.0005654 (PMC2681483; doi:10.1371/journal.pone.0005654)
Supplement: Table S2 — Primers used for in vitro real time PCR (0.06 MB DOC) [file pone.0005654.s008.doc]

| **Gene** | **Marker** | **Assay ID** |
| --- | --- | --- |
| Oct 4 | Pluripotency / mESC / germ cell | Mm00658129_gH |
| Sox2 | Pluripotency / mESC / germ cell | Mm00488369_s1 |
| Nanog | Pluripotency / mESC / germ cell | Mm02384860_g1 |
| TNAP/Alp1 | Pluripotency / mESC / germ cell | Mm00475831_m1 |
| Dnmt1 | Pluripotency/Epigenetic Regulator | Mm00599763_m1 |
| Dnmt3a | Pluripotency/Epigenetic Regulator | Mm00432870_m1 |
| Dnmt3b | Pluripotency/Epigenetic Regulator | Mm01240113_m1 |
| Blimp1 / PRDM1 | PGCs | Mm00476128_m1 |
| Fragilis / Ifitm3 | PGCs | Mm00847057_s1 |
| Stella / Dppa3 | PGCs | Mm00836373_g1 |
| cKit | PGCs | Mm00445212_m1 |
| Pum1 | PGCs | Mm00472886_m1 |
| Pum2 | PGCs | Mm00472902_m1 |
| Nanos3 | PGCs | Mm00808138_m1 |
| Vasa / Ddx4 | Meiotic GCs | Mm00802445_m1 |
| Stra8 | Meiotic GCs | Mm00486473_m1 |
| Sycp3 | Meiotic GCs | Mm00488519_m1 |
| GCNF / Nr6a 1 | Meiotic GCs | Mm00599848_m1 |
| GDF9 | Meiotic GCs (female) | Mm00433565_m1 |
| TEKT1 | Meiotic GCs (males) | Mm00495586_m1 |
| Acrosin | sperm head marker | Mm00496483_m1 |
| Tdrd1 | nudge/germinal granule form | Mm00459548_m1 |
| Bax | Apoptosis | Mm00432050_m1 |
| Bcl2 | Apoptosis | Mm00477631_m1 |
| Caspase 6 | Apoptosis | Mm00438053_m1 |
| Atg5 | Autophagy | Mm00504340_m1 |
| Beclin1 | Autophagy | Mm00517174_m1 |
| GAPDH | HOUSEKEEPING | Mm03302249_g1 |
